# Angiotensin II triggers RIPK3-MLKL-mediated necroptosis by activating the Fas/FasL signaling pathway in renal tubular cells

**DOI:** 10.1371/journal.pone.0228385

**Published:** 2020-03-05

**Authors:** Yongjun Zhu, Hongwang Cui, Jie Lv, Guojun Li, Xiaoyan Li, Feng Ye, Liangbao Zhong

**Affiliations:** 1 Department of Nephrology, the First Affiliated Hospital of Hainan Medical University, Haikou, China; 2 Department of Orthopedics, the First Affiliated Hospital of Hainan Medical University, Haikou, China; 3 The First Clinical College of Hainan Medical University, Hainan, China; Hopital Tenon, FRANCE

## Abstract

Our earlier studies proved that RIPK3-mediated necroptosis might be an important mode of renal tubular cell death in rats with chronic renal injury and the necroptotic cell death can be triggered by tumor necrosis factor-α (TNF-α) in vitro, but the triggering role of angiotensin II (AngII), which exerts notable effects on renal cells for the initiation and progression of renal tubulointerstitial fibrosis, is largely unknown. Here, we identified the presence of necroptotic cell death in the tubular cells of AngII-induced chronic renal injury and fibrosis mice and assessed the percentage of necroptotic renal tubular cell death with the disruption of this necroptosis by the addition of necrostatin-1 (Nec-1). Furthermore, the observation was further confirmed in HK-2 cells treated with AngII and RIPK1/3 or MLKL inhibitors. The detection of Fas and FasL proteins led us to investigate the contribution of the Fas/FasL signaling pathway to AngII-induced necroptosis. Disruption of FasL decreased the percentage of necroptotic cells, suggesting that Fas and FasL are likely key signal molecules in the necroptosis of HK-2 cells induced by AngII. Our data suggest that AngII exposure might trigger RIPK3-MLKL-mediated necroptosis in renal tubular epithelial cells by activating the Fas/FasL signaling pathway in vivo and in vitro.

## Introduction

Chronic kidney disease (CKD) causes serious health problems[1] and affects approximately 8–16% of adults worldwide[2, 3]. Its prognosis depends mainly on the degree of renal tubulointerstitial fibrosis (TIF) rather than glomerular damage[4]. Therefore, exploring the mechanism of TIF has great significance for the early prevention and treatment of CKD. In our earlier studies, we found that necroptosis mediated by receptor-interacting serine-threonine kinase 3 (RIP3) and mixed lineage kinase domain-like (MLKL) might play a more significant role than apoptosis in mediating the loss of renal tubular cells rather than glomerular cells death in rats subjected to subtotal nephrectomy (SNx), thus favoring the progression of TIF and CKD[5, 6].

Several studies have demonstrated that the necroptosis of tubular cells in renal injury models can be triggered by tumor necrosis factor-α (TNF-α) or other agonists [5, 7, 8]. Angiotensin II (AngII) has been recognized to exert potent effects on renal cells for the initiation and progression of renal fibrosis [9–11]. However, the role of AngII in promoting necroptosis of tubular cells has not been fully elucidated.

AngII has long been known to be the principal effector of the renin-angiotensin system and mediates kidney disease progression through its hemodynamic and nonhemodynamic effects on renal cells [12]. It has been demonstrated that AngII levels in the peritubular capillary and the proximal tubule are 1000 times higher than circulating AngII levels, suggesting that intrarenal AngII may be more important for the progression of renal injury than systemic AngII [13–15], and that human proximal tubular cells (PTECs) are sites of intrarenal AngII accumulation [16]. It has been proved that AngII can cause PTEC apoptosis and autophagy [12, 17]. However, it is still unknown whether AngII is the most common and efficient mediator of renal tubular cell necroptosis in CKD progression. In this study, we hypothesized that AngII might induce necroptosis of renal tubular epithelial cells and contributes to TIF and CKD. Our results show that AngII triggers the RIP3- and MLKL-dependent necroptosis of renal tubular epithelial cells by the Fas/FasL signaling pathway in vitro and in vivo. These data identify molecular targets for early prevention of CKD progression.

## Materials and methods

### Animals

Adult male C57BL/6 mice (purchased from Beijing Vital River Laboratory Animal Technology Co., Ltd.) were used in the study. Mice were housed in groups of five under specific pathogen-free conditions in the Hainan Research Center for drug safety evaluation and given free access to water and food. Seven- to eight-week-old male mice were randomly divided into two groups, and administered AngII (1.5 μg/kg/min; Sigma) or 0.9% sterile saline continuously using osmotic minipumps (Alzet model 2002; USA) implanted subcutaneously in the backs after uninephrectomy surgery under pentobarbital sodium anesthesia, as previously reported by Zahraa Mohammed-Ali et al.[18]. Then, all mice treated with AngII were fed 1% saline drinking water ad libitum. Simultaneously, AngII-treated mice were randomly divided into three groups (n = 6) and were injected intraperitoneally with 10% dimethyl sulfoxide (DMSO, Sigma-Aldrich, USA) or necrostatin-1 (Nec-1 1.65 mg/kg/day[6], Sigma-Aldrich, USA) or Z-VAD-FMK (zVAD, 1.0 mg/kg per day[6], MP Biomedicals, Solon, OH, USA) dissolved in 10% DMSO for 3 consecutive weeks. The AngII-treated mice were sacrificed 21 days after implantation via cervical dislocation, and blood and kidneys were collected. All experimental protocols were approved by the Institutional Ethics Committee of the First Affiliated Hospital of Hainan Medical University, and the methods were carried out in accordance with the approved guidelines.

### Renal morphological and serum analyses

Kidneys of the mice were collected when the mice were sacrificed. One portion of the kidney tissue was fixed with 4% phosphate-buffered formaldehyde and embedded in paraffin wax. To assess renal tubular morphological changes, 4-micrometer-thick sections were stained with H&E, and tubular injury was scored by assessing the tubular injury scores using a light microscope. Tubular damage was scored as follows: for tubular dilatation, 0 = no dilation and 1 = dilated tubules; for tubular atrophy, 0 = no atrophy, 1 = signs of atrophy and 2 = necroptosis, apoptosis and desquamation; for intracellular vacuoles, 0 = none, 1 = mild (<10 cells per field of view) and 2 = severe; and for hyaline deposition, 0 = no deposits and 1 = hyaline deposits. The total index score for each mouse was expressed as the mean value of all of the scores obtained. In addition, after deparaffinization and rehydration, kidney tissue sections were subjected to Masson Trichrome (Sigma-Aldrich) staining by following the manufacturers’instructions. The staining results were recorded under a bright field microscope. For the quantitative analysis, at least 10 random high-power fields (i.e., 400x) were selected and evaluated. The positive area was calculated as a % of the total area. All assessments were performed in a blinded manner by an experienced pathologist.

BUN and serum creatinine levels were determined using standard clinical biochemical techniques for assessing renal function in mice.

### Cell culture and gene expression interference

Human kidney cells (HK-2, an immortalized human proximal tubule epithelial cell line) were purchased from American Type Culture Collection (Manassas, VA, USA). The cells were cultured in DMEM/F12 medium (Gibco Life Technologies, Carlsbad, CA, USA) containing 10% FBS (HyClone, U.S.) supplemented with 1% penicillin and streptomycin (Beyotime, Shanghai, China) in a humidified incubator with 5% CO2 at 37°C. Cells were grown to 70–80% confluency and stimulated with AngII (Sigma, USA) at a concentration range of 10^−10^–10^−5^ M for 24 h and exposed to 10^−9^ M AngII for 12 h, 24 h, 36 h, 48 h, and 72 h. To further assess the necroptosis of HK-2 cells, cells were pretreated with RIP1 inhibitor (100 μM Nec-1[5]) or the pan-caspase inhibitor (10 mM zVAD[5]) for 30 min, RIPK3 blocker (0.25 μM GSK’872[19]) or MLKL inhibitor (1 μM NSA, Selleck, USA[8]) for 1 h. To elucidate the relevant mechanisms, cells were treated with an AT1R antagonist (10 μM losartan[20, 21]) and an AT2R antagonist (PD123319[20, 21]) for 30 min or with FasL inhibitor (3 μg/ml [22, 23] the neutralizing Human Fas Ligand/TNFSF6 Antibody (RD Systems, USA)) for 2 h. After pretreatment for the assigned duration, HK-2 cells were exposed to 10^−9^ M AngII for 24 h. The treated cells were collected at the assigned time for western blot analysis, TEM, and immunofluorescence staining.

### Small interfering RNA experiments

Small interfering RNAs (siRNAs) were designed and chemically synthesized (GenePharma, Suzhou, China). The following mRNA sequences for targeting RIP1, RIP3, and MLKL were used.

RIP1: 5’- GCAGUUGAUAAUGUGCAUATT-3’ (sense),

            5’- UAUGCACAUUAUCAACUGCTT-3’ (antisense),

RIP3: 5’- CCGGCUUAGAAGGACUGAATT-3’ (sense),

5’- UUCAGUCCUUCUAAGCCGGTT-3’ (antisense),

            MLKL: 5’- UCAAGGACGUGAACAGGAATT-3’ (sense),

5’- UUCCUGUUCACGUCCUUGATT-3’ (antisense).

            HK-2 cells were seeded at 70–90% confluence and transfected with the targeting siRNAs using Lipofectamine^™^ 3000 Reagent (Invitrogen Inc, USA) according to the manufacturer’s instructions for 6 h, after which cells were placed in fresh medium for 24 h. Cells transfected with nonspecific siRNA served as controls. Then, the cells were stimulated with 10^−9^ M AngII. Twenty-four hours later, the cells were harvested for western blot analysis to determine the transfection efficiency.

### Flow cytometric analysis

Quantitation of necrotic cells in different groups was performed using a FITC annexin V apoptosis detection kit (BD Biosciences, San Diego, CA) according to the manufacturer’s protocol. Briefly, the cells were seeded into 6-well plates at a density of 5 × 10^5^ cells/well and exposed to AngII at different concentrations for different times. Then, the cells were harvested, washed twice with precooled PBS and resuspended in 100 μl of binding buffer. After the addition of 5 μl of FITC annexin V and PI, the cells were incubated for 15 min at room temperature in the dark. The samples were diluted with 400 μl of binding buffer and analyzed on a flow cytometer (BD Biosciences, San Jose, CA). The percentages of the cells residing in the upper right (necrotic cells, annexin V^+^/PI^+^) and the lower right (apoptotic cells, annexin V^+^/PI^-^) regions of the annexin V-FITC scatter plots were calculated.

### Western blot analysis

Western blotting was performed in accordance with the manufacturer’s instructions. The kidney tissue specimens and HK-2 cells were frozen in liquid nitrogen and kept at −80°C, followed by homogenization or lysis in ice-cold radioimmunoprecipitation assay (RIPA) buffer (Beyotime, Nantong, Jiangsu, China). The total protein concentration was determined using the Enhanced Bicinchoninic Acid (BCA) Protein Assay Kit (Beyotime, Jiangsu, China). The extracted protein was mixed with 5× sodium dodecyl sulfate (SDS)-polyacrylamide gel electrophoresis (PAGE) sample loading buffer, boiled for 5 min, and separated via SDS-PAGE, followed by transfer onto a polyvinylidene difluoride (PVDF) membrane (EMD Millipore, Billerica, Massachusetts, USA). After blocking nonspecific binding sites with a 5% nonfat dry milk solution, the membranes were incubated overnight at 4°C with the following primary antibodies: anti-RIP monoclonal antibody (#3493, Cell Signaling Technologies, Danvers, MA, USA; 1:1000 dilution), anti-phospho-RIP polyclonal antibody (#31122, Cell Signaling Technologies, Danvers, MA, USA; 1:400 dilution) or anti-phospho-RIP monoclonal antibody (#65746, Cell Signaling Technologies, Danvers, MA, USA; 1:400 dilution), anti-RIP3 monoclonal antibody (#95702, Cell Signaling Technologies, Danvers, MA, USA; 1:1000 dilution) or anti-RIP3 polyclonal antibody (ab152130, Abcam, Cambridge, MA, USA; 1:1000 dilution), anti-phospho-RIP3 monoclonal antibody (ab205421, Abcam, Cambridge, MA, USA; 1:400 dilution), anti-MLKL polyclonal antibody (#28640, Cell Signaling Technologies, Danvers, MA, USA; 1:1000 dilution) or anti-MLKL monoclonal antibody (#14993, Cell Signaling Technologies, Danvers, MA, USA; 1:1000 dilution), anti-phospho-MLKL monoclonal antibody (ab208910, Abcam, Cambridge, MA, USA; 1:400 dilution) or anti-phospho-MLKL monoclonal antibody (#91689, Cell Signaling Technologies, Danvers, MA, USA; 1:400 dilution), anti-Fas polyclonal antibody (sc-1023, Santa Cruz Biotechnology, CA, USA; 1:1000 dilution), anti-Fas ligand polyclonal antibody (ab15285, Abcam, Cambridge, MA, USA; 1:1000 dilution) and anti-ß-actin monoclonal antibody (sc-47778, Santa Cruz Biotechnology, CA, USA; 1:1000 dilution). Subsequently, the membranes were incubated with goat anti-rabbit IgG and then visualized using an ECL detection kit (Beyotime, Nantong, Jiangsu, China). The protein bands of interest were visualized using an ECL detection kit (Beyotime, Nantong, Jiangsu, China). The intensity of the bands was analyzed using Image Lab version 2.1 (Bio-Rad). The quantitative densitometric values for each protein were normalized to ß-actin.

### Transmission electron microscopy (TEM)

Kidney tissue and cells treated with reagents as shown previously were harvested, 1-mm^3^ renal tissue fragments and centrifuged cells were fixed in 4% glutaraldehyde phosphate buffer (pH 7.4) overnight at 4°C, rinsed with PBS and postfixed in 2% osmium tetroxide. Then, the fixed renal tissue fragments and cells were dehydrated in an ascending series of ethanol and embedded in epoxy resin. Finally, ultrathin sections (60–70 nm) were stained with uranyl acetate and alkaline lead citrate, and the cells were visualized under a transmission electron microscope (TEM) (Hitachi-7700, Japan).

### Immunofluorescence detection of RIP-3 and in situ fluorescent TUNEL staining

Sagittal kidney tissue sections (4 μm thick) and HK-2 cells seeded on chamber slides (Thermo Scientific) and incubated with treatments as described previously were prepared for immunofluorescence staining and in situ fluorescent TUNEL staining. First, the sections and cells were fixed with 4% paraformaldehyde (Sigma-Aldrich), followed by permeabilization in 0.1% Triton X-100 and incubated with 5% BSA (Sigma-Aldrich). The slides and cells were then incubated with anti-RIP3 monoclonal antibody (#95702, Cell Signaling Technologies, Danvers, MA, USA; 1:100 dilution) or anti-RIP3 polyclonal antibody (ab152130, Abcam, Cambridge, MA, USA; 1:100 dilution) overnight at 4°C and then incubated with Alexa Fluor 555-conjugated goat anti-rabbit IgG (H+L) (#4413, Cell Signaling Technologies, Danvers, MA, USA; 1:200 dilution). After rinsing 3 times in 0.1 M PBS (pH 7.4), the samples were incubated with in situ cell death detection kit reagents (Fluorescein, Roche, Basel, Switzerland) according to the manufacturer’s instructions and counterstained with 4',6-diamidino-2-phenylindole (DAPI). Finally, the images were captured by confocal microscopy (LEICA TCS SP2, Wetzlar, Germany), and the cell counting was performed by a pathologist blinded to the experimental conditions.

### Statistical analysis

Data are presented as the means ± standard errors of the mean (S.E.M.s). SPSS 17.0 statistical software (SPSS Inc., IL, USA) was used for statistical analyses. Multiple group comparisons were performed via an ANOVA followed by a least significant difference (LSD) or Dunnett’s post hoc test. P<0.05 was considered statistically significant.

## Results

### Nec-1 improves renal functions and renal pathological lesions in AngII-induced chronic renal injury and fibrosis mice

To test whether Nec-1 would alleviate kidney damage caused Ang II, an animal model with properties of progressive renal tubulointerstitial fibrosis and CKD was established through continuous infusion of AngII (1.5μg/kg/min) using osmotic minipumps implanted subcutaneously in the backs of C57BL/6 mice that had undergone uninephrectomy. The mice were then randomly divided into the Ang II-treated group, which only received a continuous Ang II infusion, the Ang II + Nec-1 treated group, which received continuous infusion of Ang II and intraperitoneal administration of RIP1 inhibitor(Nec-1, 1.65 mg/kg/d), and the Ang II + zVAD treated group, which received continuous infusion of Ang II along with intraperitoneal administration of the pan-caspase inhibitor (zVAD, 1.0 mg/kg/d). The mice from the control group did not receive any treatment. The mice were then killed to collect blood samples and kidneys on day 21 after infusion with AngII. The renal function and hematoxylin and eosin (H&E) and Masson Trichrome staining of kidney tissue from the mice were assessed.

**Fig 1:**
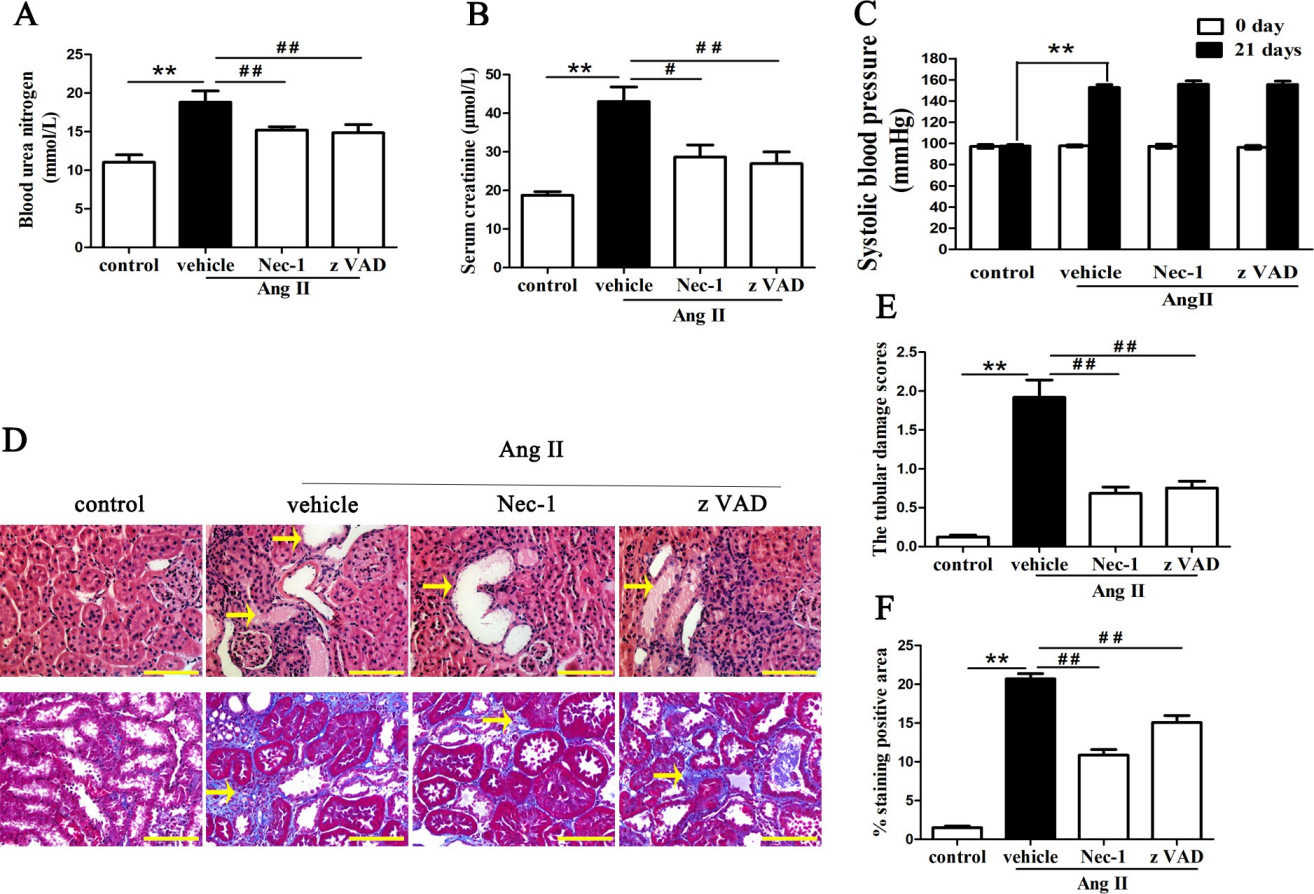
Effect of Nec-1 on renal function, blood pressure, and renal tubule injury in Ang II-induced chronic renal injury mice. The levels of BUN (A) and Scr (B) were higher in AngII-treated mice than in control mice, and administration of Nec-1 or zVAD significantly attenuated renal dysfunction of model mice. (C) Systolic blood pressure was elevated to the similar level between three treatment groups. (D) Representative images of H&E-stained and Masson trichrome-stained mouse renal tubulointerstitial lesions in AngII-treated mice treated with or without Nec-1 or zVAD. The tubular injury scores (E) and Masson-positive interstitial collagen regions (F) were analyzed under a light microscope. Data represent the mean of three independent experiments ± S.E.M. N = 6 mice per group, ** p<0.01 compared with the control group, ^#^p<0.05 compared with the vehicle group, ^##^p<0.01 compared with the vehicle group.

As shown in Fig 1A and 1B, the levels of blood urea nitrogen (BUN) and serum creatinine (Scr) were significantly higher in the AngII-treated mice than in the control mice, which indicated the animal model of Ang II-induced chronic renal injury was successfully established. Following Nec-1 or zVAD administration, the levels of BUN (Fig 1A) and Scr (Fig 1B) in the AngII-treated mice decreased. In addition, Ang II treatment increased systolic blood pressure in AngII-treated mice, but Nec-1 and zVAD could not decrease the increased blood pressure of the AngII-treated mice (Fig 1C).

We further examined the histopathologic features of the kidney tissues retrieved from the animal model experiments. Masson Trichrome staining revealed that Ang II treatment led to a significant increase in interstitial collagen deposition in the kidney, compared with the control group (p < 0.01) (Fig 1D and 1F). However, such renal fibrotic response was suppressed partly in the Nec-1- or zVAD-treated mice with chronic Ang II infusion (Fig 1D). More importantly, from the H&E-stained kidney tissue section (Fig 1D), we clearly found that the morphology of the renal tubules in the AngII-treated mice exhibited severe and widespread structural damage, including tubular dilatation, vacuolar degeneration, atrophy, desquamation of epithelial cells, and hyaline deposition in the tubular lumen. Furthermore, the tubular injury scores were significantly higher than those of the control group, and the tubular pathological lesions were improved in mice treated with Nec-1 or zVAD (Fig 1E).

### Nec-1 prevents the necroptosis of renal tubular epithelial cells in Ang II-induced chronic renal injury model

**Fig 2:**
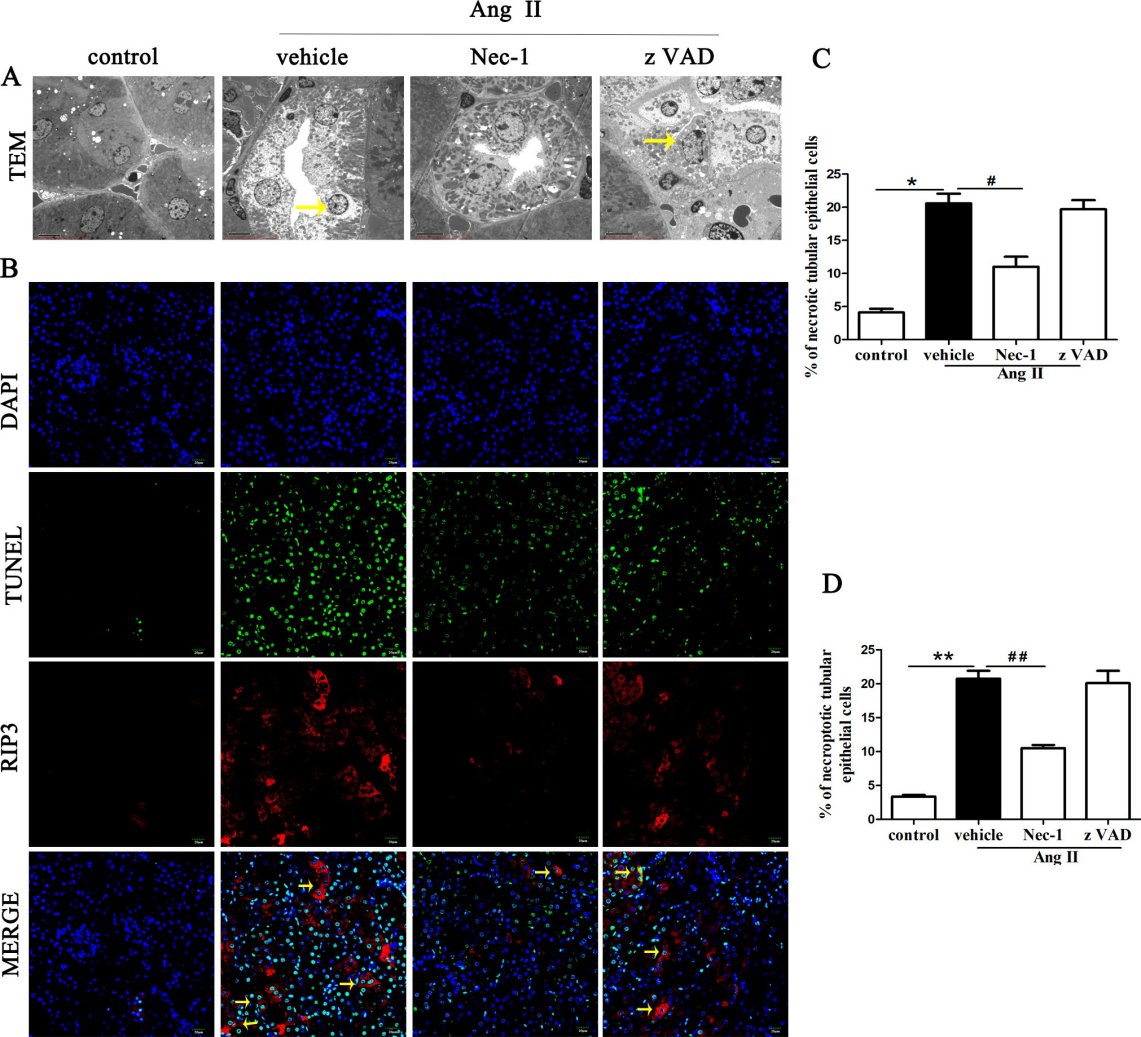
Effect of Nec-1 on necroptosis of renal tubular epithelial cells in Ang II-induced renal injury model (A) Representative images of necroptotic tubular epithelial cells with typical necrotic morphological features under TEM in the kidney tissue of the Ang II-induced renal injury mice treated with or without Nec-1 or zVAD. Scale bars represent 5 μm (B) TUNEL-stained (green fluorescence) cells in kidney tissue from Ang II-induced renal injury mice treated with or without Nec-1 or zVAD were costained to detect RIP3 (red fluorescence) and the nucleus (DAPI, blue fluorescence). Scale bars represent 20 μm. (C) The percentage of necrotic tubular epithelial cells were analyzed in the Ang II-induced chronic renal injury mice treated with or without Nec-1 or zVAD. (D) The data are presented as the % ratio of necroptotic tubular epithelial cells in the Ang II-induced chronic renal injury mice treated with or without Nec-1 or zVAD. Data represent the mean of three independent experiments ± S.E.M. N = 6 mice per group, *p<0.05 compared with the control group, **p<0.01 compared with the control group, ^#^p<0.05 compared with the vehicle group, ^##^p<0.01 compared with the vehicle group.

To further elucidate the morphological characteristics of renal tubular epithelial cells in a mouse model of Ang II-induced chronic renal injury, transmission electron microscopy (TEM) was used to observe the ultrastructure of tubular epithelial cells. As shown in Fig 2A, many of the tubular epithelial cells in the AngII-treated mice displayed a necrotic morphology, showing marked expansion of the cell volume, plasma membrane rupture, organelle disappearance and loss of cell contents, consistent with the typical morphological features of necrotic cell death. The apoptotic tubular epithelial cells showing cells shrinkage, nuclear condensation and other characteristic features in the AngII-treated mice were occasionally found. Furthermore, the percentage of necrotic cell death was significantly elevated in the AngII-treated mice (p < 0.05, Fig 2C). Pretreatment with Nec-1 led to better preservation of the integrity of the renal tubular epithelial cells, and the percentage of necrotic cell death was markedly lower than that in the AngII-treated mice (Fig 2A and 2C), but zVAD did not significantly decreased the percentage of necrotic cell death in the AngII-treated mice. Interestingly, we observed that necrotic cell death appears in renal tubular cells rather than glomerular parenchymal cells in renal tissue from AngII-induced renal injury and fibrosis mice under TEM.

To further confirm that the necrotic tubular epithelial cells observed by TEM were necroptotic cells, fluorescent TUNEL staining and RIP3 immunostaining assays were performed. By confocal microscopy, we observed that RIP3 expressed mainly in tubular cells of the kidney, especially in the cytoplasm of proximal tubular epithelial cells (Fig 2B). However, RIP3 protein were not detected, or only scarcely detected, in glomeruli. Furthermore, we found that the percentage of TUNEL-positive, RIP3-positive tubular epithelial cells (necroptotic renal tubular epithelial cells) was significantly higher in the AngII-treated mice (p < 0.01, Fig 2D) than in the control mice. The necroptotic cell percentage significantly decreased when RIP1 were blocked, but zVAD could not significantly decreased the necroptotic cell percentage in the AngII-treated mice (Fig 2D), which is consistent with the TEM results. This finding further confirms that necroptosis of renal tubular epithelial cells might play a significant role on tubular epithelial cells death in AngII-induced chronic renal injury mice and AngII might be involved in necroptosis of renal tubular epithelial cells.

### Nec-1 suppresses necroptosis-related proteins expression in Ang II-induced chronic renal injury model

During the necroptotic process, RIP3, MLKL and their phosphorylated proteins are the key proteins of the necroptosis pathway, and their expression levels have been shown to correlate closely with necroptosis[24]. RIP3 is activated by binding and interacting with RIP1, and activated RIP3 recruits and subsequently phosphorylates downstream signaling molecule MLKL (on Ser345/Ser347 in mouse MLKL)[25–28]. MLKL phosphorylation is believed to trigger a molecular switch for programmed cell death, and result in necroptosis.

**Fig 3:**
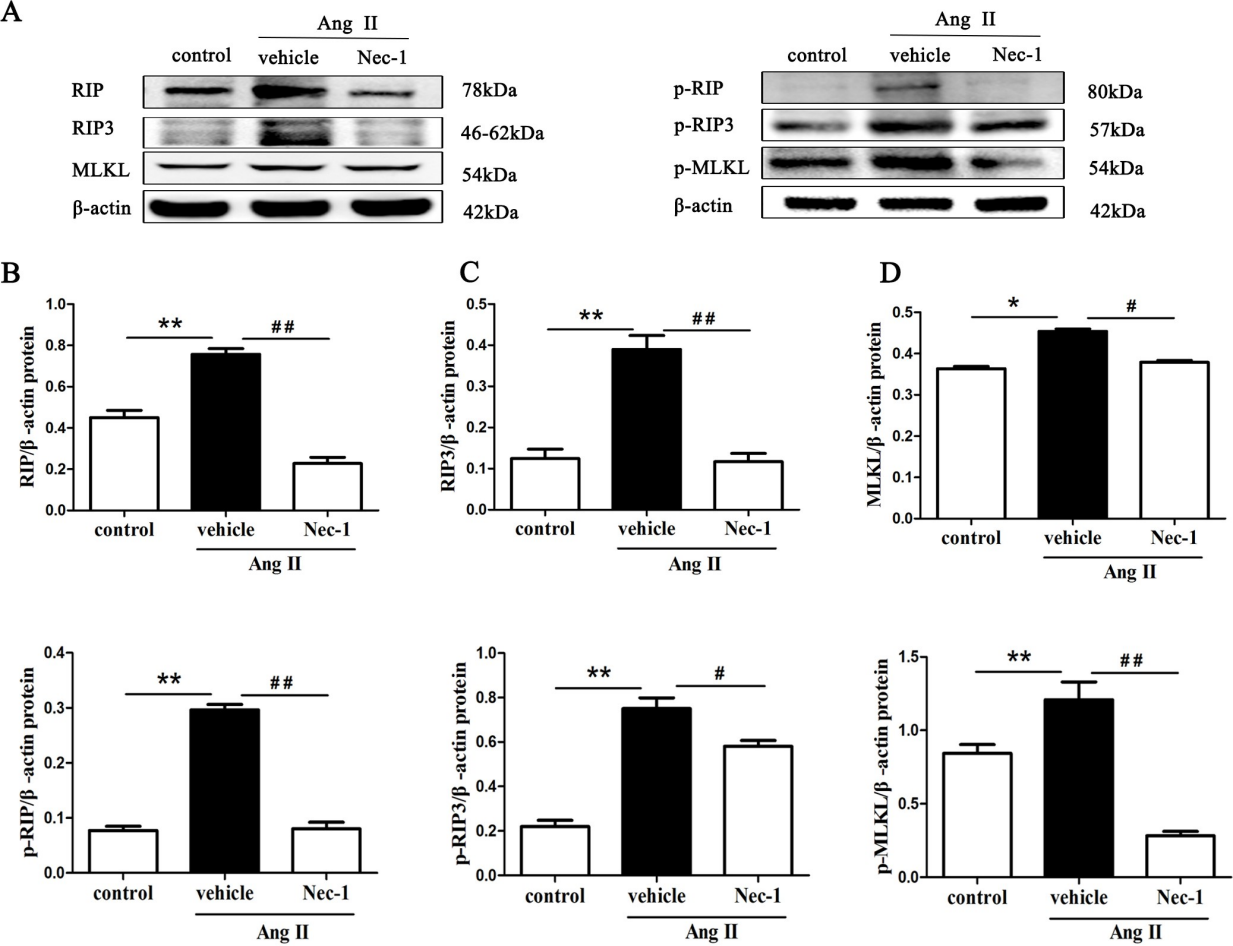
Nec-1 suppresses RIP and p-RIP, RIP3 and p-RIP3, MLKL and p-MLKL protein expression in the kidney tissues from AngII-treated mice (A) Representative blots of RIP and p-RIP, RIP3 and p-RIP3, MLKL and p-MLKL protein in the Ang II-induced chronic renal injury mice are shown. Western blot analyses of RIP and p-RIP (B), RIP3 and p-RIP3(C), MLKL and p-MLKL (D) protein expression in the kidney tissue of Ang II-induced renal injury mice treated with or without Nec-1 or zVAD. Data represent the mean of three independent experiments ± S.E.M. N = 6 mice per group, **p<0.01 compared with the control group, ^#^p<0.05 compared with the vehicle group, ^##^p<0.01 compared with the vehicle group.

In this study, we found that the levels of RIP and its phosphorylated forms (phospho-RIP, p-RIP), RIP3 and phospho-RIP3 (p-RIP3), MLKL and phospho-MLKL (p-MLKL)) in the kidney tissues from AngII-treated mice were elevated, and the RIP1 stabilizer (Nec-1) could partially inhibit the above protein expression in the animal models (Fig 3).

Together, these results imply that tubular epithelial cell necrosis in AngII-treated mice under TEM may involve the RIPK3/MLKL-dependent necroptosis pathway and might be related to AngII.

#### AngII induces RIP3/MLKL-dependent necroptosis of HK-2 cells in vitro

**Fig 4:**
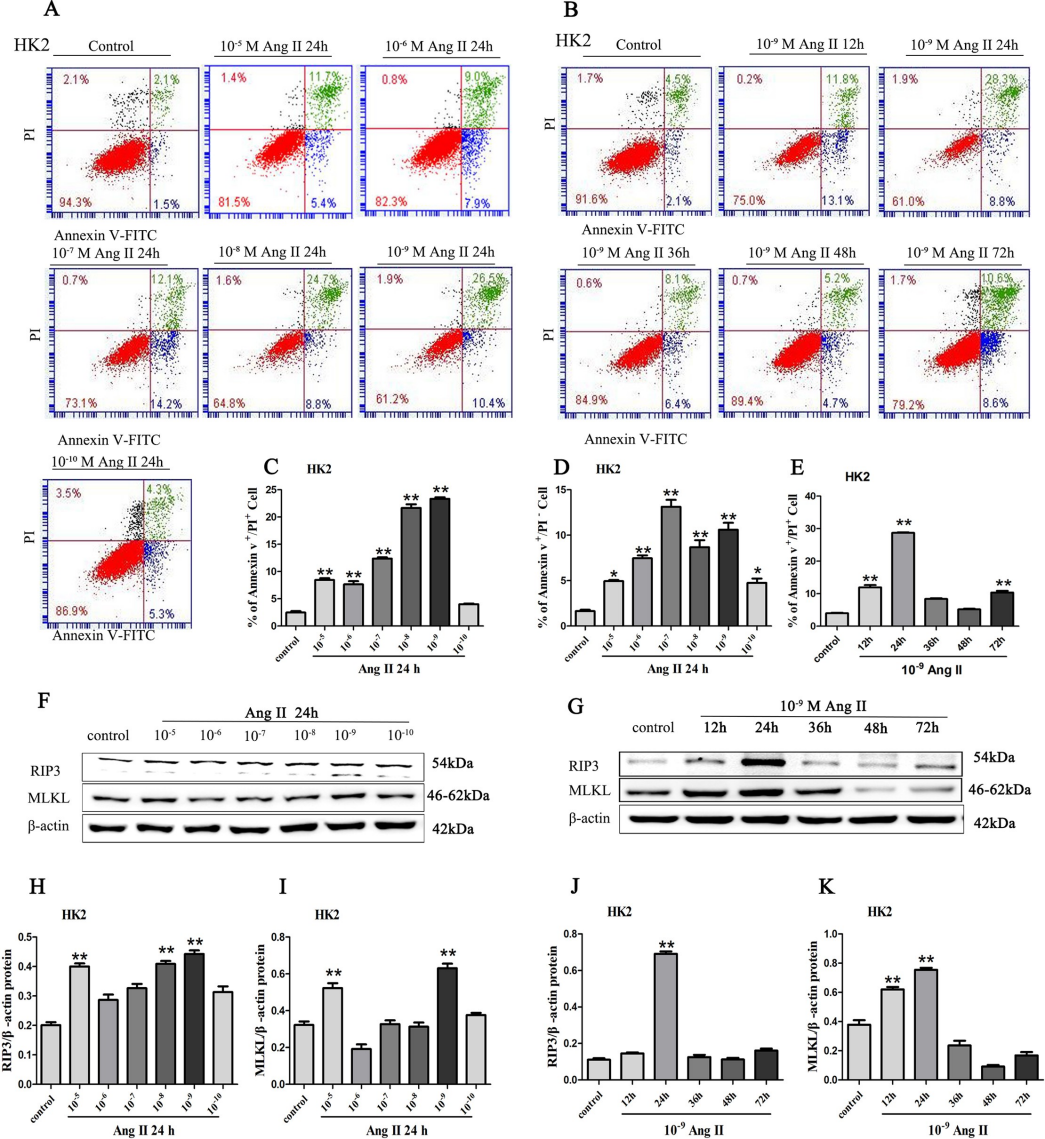
The assessment of necroptosis and the expression levels of p-RIP3 and p-MLKL in HK-2 cells treated with AngII. (A) HK-2 cells were treated with varying concentrations of AngII (10^−10^–10^−5^ M) for 24 h. (B) HK-2 cells were exposed to 10^−9^ M AngII for different times. After treatment, HK-2 cells were stained with annexin V-FITC and PI to determine necrotic cells (annexin V^+^/PI^+^ cells) and apoptotic cells (annexin V^+^/PI^-^ cells) using a flow cytometry assay. The bar chart shows that the ratio of annexin V^+^/PI^+^ cell numbers was highest in the HK-2 cells treated with 10^−9^ M AngII for 24 h (C, E) and the ratio of annexin V^+^/PI^-^ cells (apoptotic cells) numbers was highest in the HK-2 cells treated with 10^−7^ M AngII (D) for 24 h. Protein expression of RIP3 and MLKL was determined by western blotting of the total proteins isolated after stimulation of HK-2 cells with varying concentrations of AngII for 24 h (F, H, I). Protein expression of RIP3 and MLKL was determined by western blotting of the total proteins isolated from HK-2 cells exposed to 10^−9^ M AngII for different times (G, J, K). Data represent the mean of three independent experiments ± S.E.M. with n = 3, *p<0.05 compared with the control group, **p<0.01 compared with the control group.

With in vivo experiments, we demonstrated that necroptosis of renal tubular epithelial cells might play a significant role on tubular epithelial cells excessive death in AngII-induced chronic renal injury mice and AngII might be involved in necroptosis of renal tubular epithelial cells. Therefore, HK-2 cells were exposed to varying concentrations of AngII (10^−10^–10^−^_5_ M, Fig 4A) for different times (12, 24, 48 or 72 h, Fig 4B) for further support of our observations in vivo. The results of flow cytometric analysis showed a significant increase in the percentage of annexin V^+^/PI^+^ cells (necrotic cells) in the cells treated with 10^−^_9_ M AngII for 24 h compared to that of the control group and the other treated groups (Fig 4A, 4B, 4C and 4E). Again, the data from western blot analysis showed a sharp increase in RIP3 and MLKL expression in the HK-2 cells treated with 10^−^_9_ M AngII for 24 h (Fig 4F–4K). Thus, AngII is associated with necrotic tubular cells mediated by RIP3 and MLKL in a specific spatiotemporal manner. In addition, we also found that a significant increase in the percentage of annexin V^+^/PI^-^ cells (apoptotic cells) in the cells treated with 10^−^_7_M AngII compared to that of the control group and the other treated groups (Fig 4A and 4D).

#### RIP1/3 and MLKL inhibition blocks AngII-induced necroptosis in vitro

**Fig 5:**
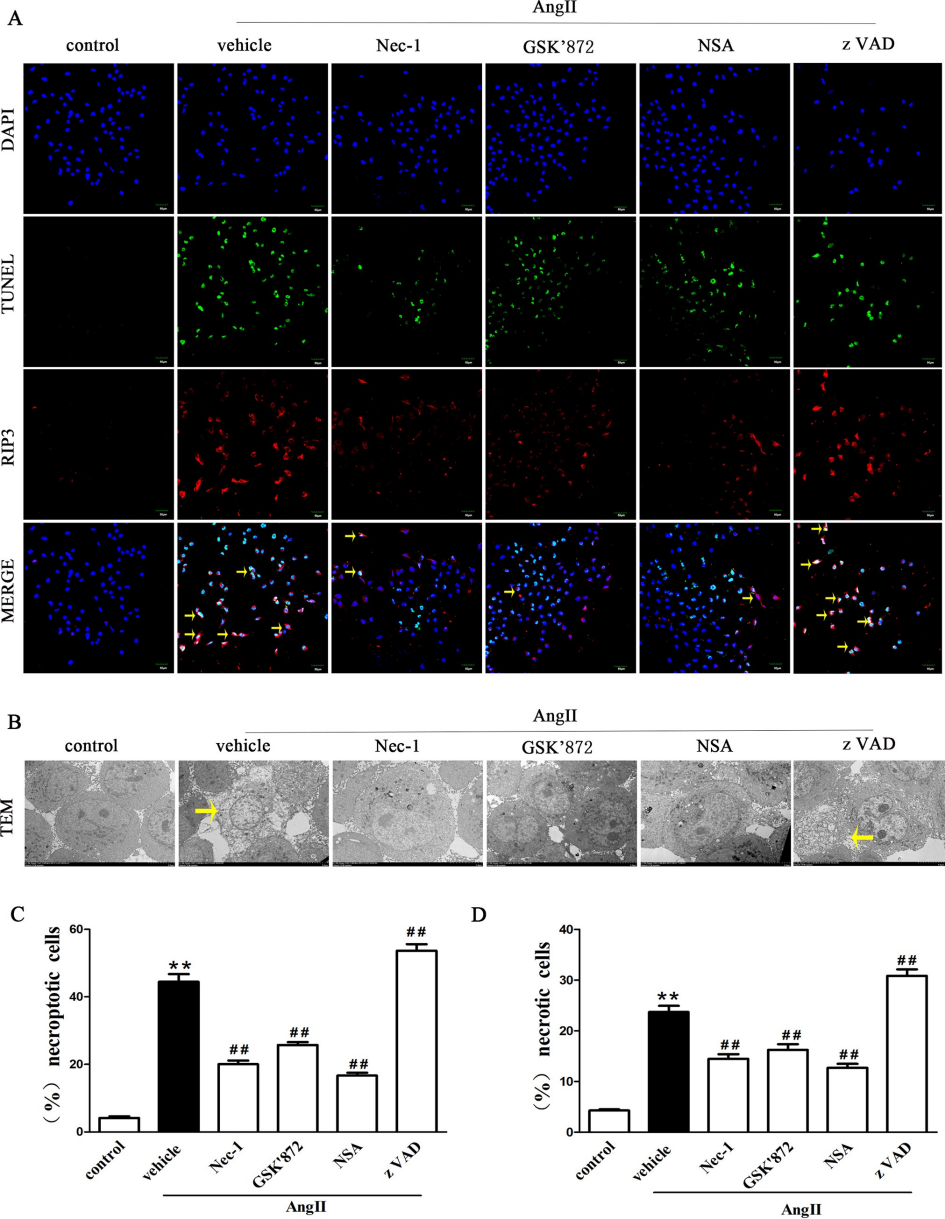
Disruption of RIP1, RIP3, and MLKL suppresses the necroptosis of HK-2 cells induced by Ang II (**A**) TUNEL-stained (green fluorescence) HK-2 cells in each treatment group were costained to detect RIP3 (red fluorescence) and the nucleus (DAPI, blue fluorescence). Scale bars represent 50 μm. (B) Representative images of necroptotic HK-2 cells (yellow arrow) were observed under TEM. Scale bars represent 5 μm. **(C)** The data are presented as the % ratio of necroptotic cells among HK-2 cells treated with 10^−9^ M AngII for 24 h. **(D)** The data are presented as the % ratio of necrotic cells among HK-2 cells treated with 10^−9^ M AngII for 24 h. Data represent the mean of three independent experiments ± S.E.M. with n = 3, **p<0.01 compared with the control group, ^#^p<0.05 compared with the vehicle group, ^##^p<0.01 compared with the vehicle group.

We evaluated the necroptotic incidence of AngII-induced HK-2 cells via fluorescent TUNEL staining and RIP3 immunofluorescence assays to further investigate the role of AngII in necroptotic HK-2 cell. As shown in Fig 5A, TUNEL was found in the nucleus of cells, and RIP3 localized particularly in the cytoplasm of the HK-2 cells. The percentage of TUNEL-positive, RIP3-positive HK-2 cells was significantly higher in the group of HK-2 cells stimulated with AngII than in the control group (Fig 5C). Importantly, prestimulation with Nec-1, GSK’872 (a RIPK3 blocker) or necrosulfonamide (NSA, a MLKL inhibitor) significantly decreased the percentage of TUNEL-positive, RIP3-positive HK-2 cells (Fig 5C). However, pretreatment with zVAD elevated the percentage of TUNEL-positive, RIP3-positive HK-2 cells (Fig 5C). These results further demonstrated that AngII can induce RIP3- and MLKL-dependent necroptosis of tubular epithelial cells in vitro.

TEM also showed the morphological changes in tubular epithelial cells treated with 10^−^_9_ M AngII for 24 h. We found that injured epithelial cells often exhibited ultrastructural signs of necrosis, such as loss of electron density, ballooning of the mitochondria, nuclear chromatin irregularities, outer membrane disruption, and cytoplasm and organelle expulsion (Fig 5B), and the percentage of necrotic cells increased significantly (Fig 5D). Furthermore, pretreatment with Nec-1, GSK’872 (a RIP3 inhibitor), or NSA (an MLKL inhibitor) markedly decreased the percentage of necrotic cells among HK-2 cells treated by AngII. In addition, we found that pretreatment with zVAD elevated the percentage of necrotic HK-2 cells.

**Fig 6:**
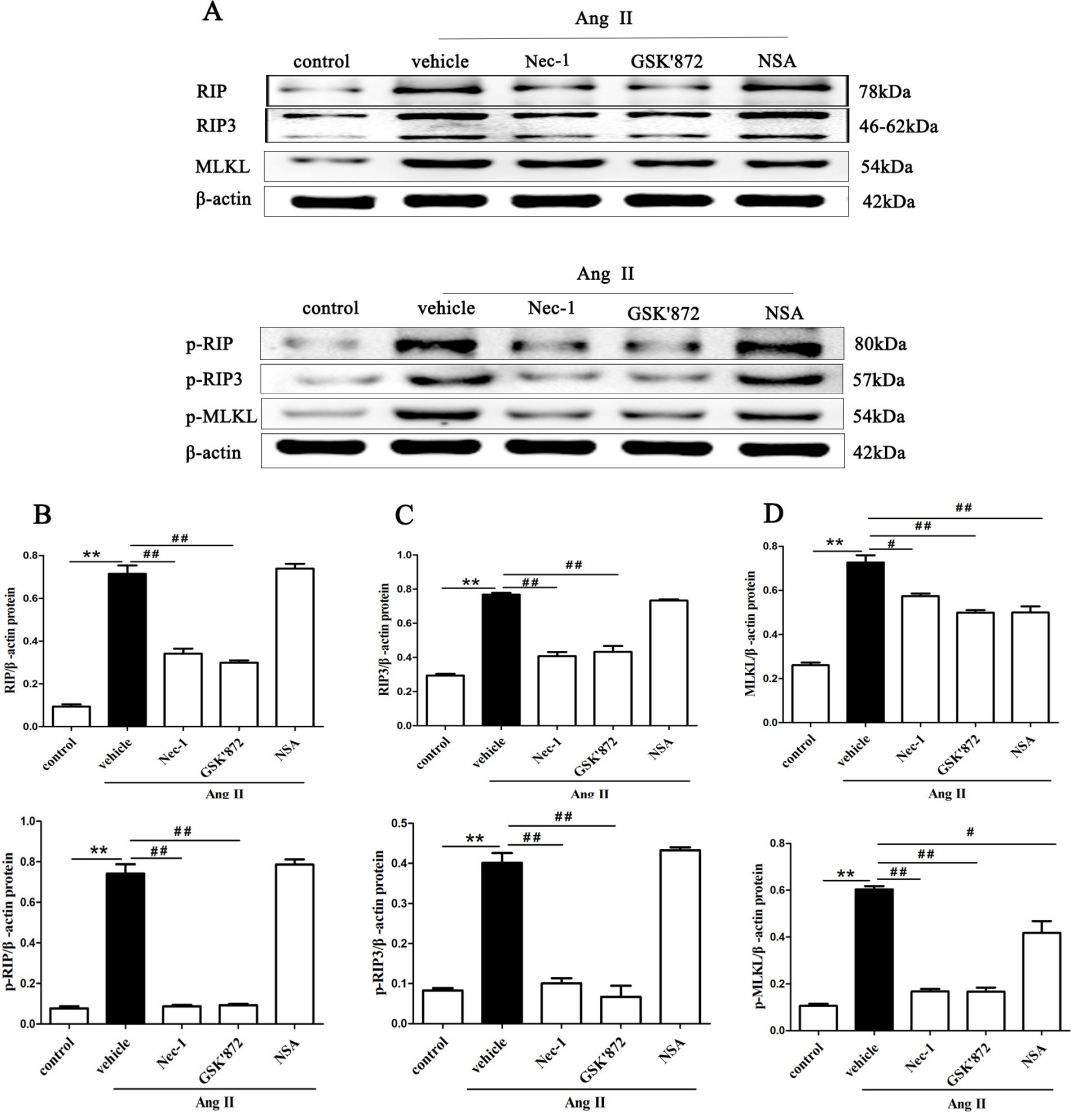
Disruption of RIP1, RIP3, and MLKL blocks necroptosis-related key proteins expression in Ang II-induced HK-2 cells. HK-2 cells were pretreated with a RIP1 inhibitor (Nec-1), a RIP3 inhibitor (GSK’872), and an MLKL inhibitor (NSA) before being exposed to AngII. The levels of RIP and p-RIP, RIP3 and p-RIP3, MLKL and p-MLKL in HK-2 cells were detected by western blotting after treatment with AngII for 24 h. (**A**) Representative blots are shown. Western blot analyses for RIP and p-RIP (**B**), RIP3 and p-RIP3 (**C**), MLKL and p-MLKL (**D**) protein expression in HK-2 cells in each treatment group are shown. Data represent the mean of three independent experiments ± S.E.M. with n = 3, **p<0.01 compared with the control group, ^#^p<0.05 compared with the vehicle group, ^##^p<0.01 compared with the vehicle group.

To confirm the phenotype observed with TEM is associated with RIP3, MLKL protein and their phosphorylated forms, the protein expression of RIP and p-RIP (Fig 6A and 6B), RIP3 and p-RIP3(Fig 6A and 6C), MLKL and p-MLKL(Fig 6A and 6D) in HK-2 cells treated with AngII was determined by western blotting. After pretreatment with Nec-1 or GSK’872, the levels of RIP, RIP3 and MLKL and their phosphorylated forms in HK-2 cells treated with 10^−^_9_ M AngII for 24 h was dramatically decreased, and MLKL and p-MLKL protein expression was significantly downregulated in cells pretreated with NSA. The data further confirmed that AngII exposure might leads to necroptosis of renal tubular epithelial cells in vitro.

#### Fas and FasL could be the target signal molecule of AngII in the AngII-induced necroptosis of renal tubular epithelial cells

**Fig 7:**
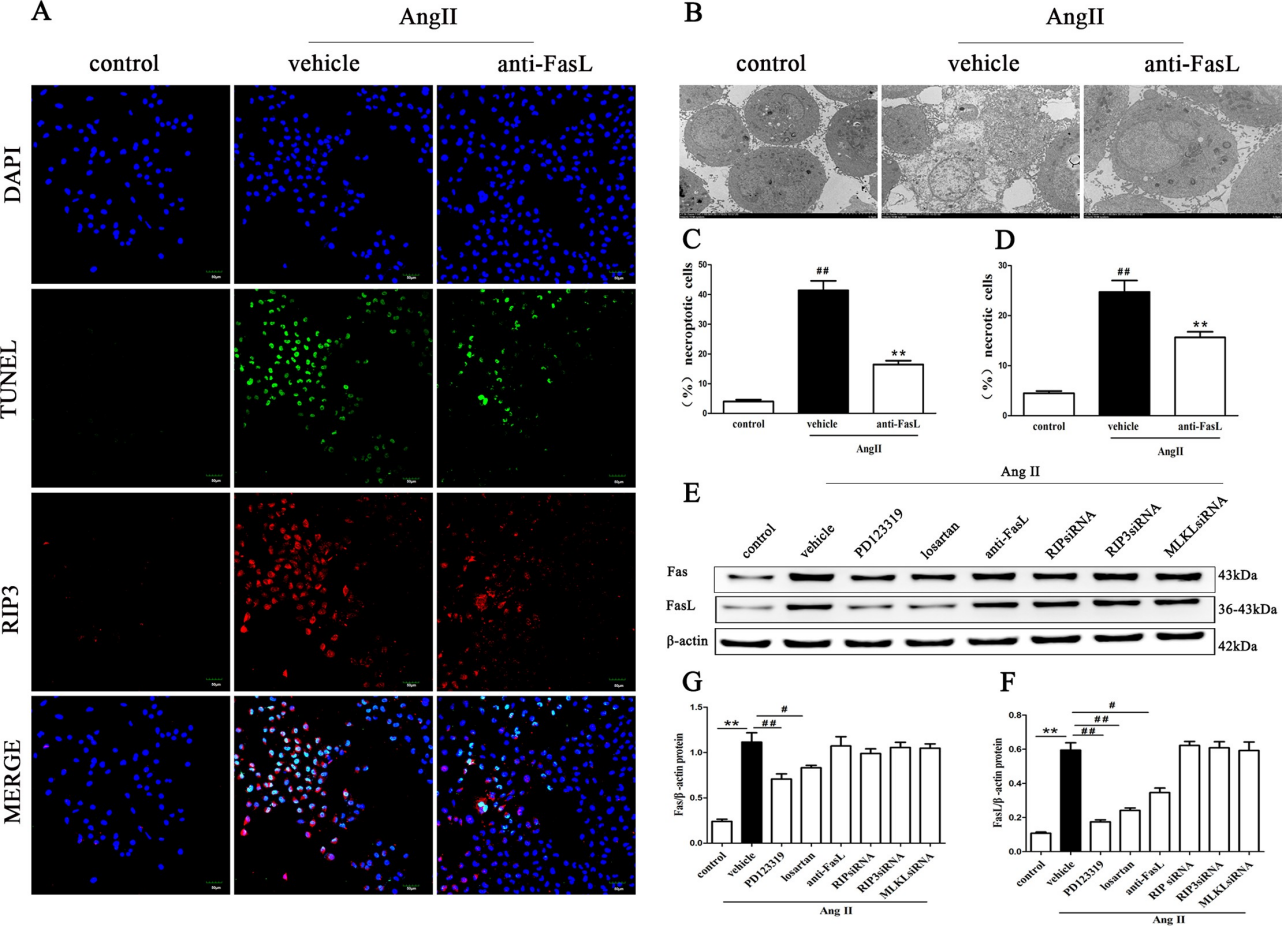
AngII-induced necroptosis in HK-2 cells requires Fas/FasL. HK-2 cells were pretreated with FasL inhibitor (the neutralizing Human Fas Ligand/TNFSF6 Antibody) before exposure to 10^−9^ M AngII. Necroptotic cells were assessed by fluorescence staining and a TEM assay 24 h later. **A** shows representative images of immunofluorescence staining for RIP3 (red fluorescence) and in situ fluorescence TUNEL staining (green fluorescence). Scale bars represent 50 μm. B shows representative TEM images. Cell death was assessed by quantifying the % ratio of TUNEL- and RIP3-positive cells (C) and the % ratio of necrotic HK-2 cells (D). Scale bars represent 5 μm. HK-2 cells were pretreated with PD123319, losartan, and a FasL inhibitor and transfected with specific small interfering (si) RNA for RIP1, RIP3 and MLKL before being exposed to AngII. The protein expression of Fas and FasL was determined by western blotting for HK-2 cells treated with AngII (E, G, F). Data represent the mean of three independent experiments ± S.E.M. with n = 3, **p<0.01 compared with the control group, ^#^p<0.05 compared with the vehicle group, ^##^p<0.01 compared with the vehicle group.

To explore which protein could be the target signal protein for AngII, we studied the role of Fas and FasL in the necroptosis of HK-2 cells using immunofluorescence detection of RIP3 and in situ fluorescence TUNEL staining and TEM. As shown in Fig 7A–7D, the percentage of TUNEL-positive, RIP3-positive HK-2 cells found with confocal microscopy (Fig 7A and 7C) and the percentage of necrotic cells found with TEM (Fig 7B and 7D) were lower when the FasL signal molecule was blocked with the neutralizing Human Fas Ligand/TNFSF6 Antibody than in the condition treated with AngII alone.

We further determined the expression levels of Fas and FasL protein in HK-2 cells exposed to AngII. When the cells were treated with 10^−^_9_ M AngII for 24 h, we found that AngII exposure significantly increased Fas and FasL protein expression (Fig 7E and 7F). Importantly, disruption of AT1R with losartan(an AT1R antagonist) and disruption of AT2R with PD123319 (an AT2R antagonist) significantly reduced the levels of Fas and FasL protein in HK-2 cells (Fig 7E and 7F), indicating that Fas and FasL were likely the target downstream signaling molecules of AngII. However, the expression of Fas and FasL did not change very significantly after transfection with the respective specific small interfering (si) RNA for RIP1, RIP3 and MLKL before being exposure to AngII (Fig 7E and 7F), indicating that Fas and FasL were likely to be target signaling molecules downstream of AngII and upstream of RIP1, RIP3 and MLKL.

These data confirm that Fas and FasL serve as the target signal molecule of AngII and its receptors and mediate the renal tubular cell necroptosis induced by AngII.

## Discussion

In our previous study, we showed that necroptotic cell death is a more significant cause of the loss of renal tubular cells in SNx rats than apoptosis [5]. This discovery raises the question of which factors and distinct signaling pathways can trigger the necroptosis of renal tubular cells in progression of chronic renal damage. In the present study, we demonstrated that AngII might effectively trigger the necroptosis of renal tubular epithelial cells in AngII-induced chronic renal injury mice and that Nec-1, the RIP1 stabilizer, significantly reduced the percentage of necroptotic cells and attenuated tubulointerstitial lesion in AngII-treated mice.

A large amount of evidence indicates that AngII exerts key and adverse nonhemodynamic effects on renal cells in the initiation and progression of renal fibrosis and CKD [29–31]. The underlying mechanisms involved in AngII-induced chronic renal injury and fibrosis are incompletely understood. Recent studies have shown that AngII can lead to PTEC apoptosis, growth, and differentiation[17] and that PTECs are key targets regulated by AngII [32]. In the present study, we found that AngII exposure led to renal tubular epithelial cell necroptosis and tubulointerstitial lesion. Importantly, the RIP1 stabilizer (Nec-1) abrogated the necroptotic cell death and renal pathological injury triggered by AngII cytotoxicity in an AngII-induced renal injury and fibrosis mouse model. The results indicate that that AngII-induced necroptosis plays pathological important role on renal tubular epithelial cell excessive death in the development of chronic renal injury and fibrosis. In addition, Ang II treatment increased blood pressure in AngII-treated, AngII+Nec-1 and AngII+zVAD treated mice with no differences between the three treatment groups. Therefore, we speculate that Ang II exposure led to the development of renal interstitial fibrosis because Ang II might lead to renal tubular epithelial necroptosis except increasing systemic blood pressure.

Interestingly, we observed that necroptotic cell death appears in renal tubular cells rather than glomerular parenchymal cells in the TEM and immunofluorescence staining images of renal tissue from AngII-induced chronic renal injury and fibrosis mice, likely because PTECs are a key site of AngII action, providing evidence supporting the ideas of Ana Konvalinka et al. [12]. Furthermore, when the HK-2 cells were treated with 10^−^_9_ M AngII for 24 h, we also found that AngII exposure significantly increased the percentage of necroptotic cells and elevated the levels of marker proteins of necroptosis (such as RIP, RIP3 and MLKL). It was more important that the level of p-RIP, p-RIP3 and p- MLKL protein (activated forms of RIP, RIP3 and MLKL) were also elevated in AngII-induced HK-2 cells. In addition, we observed that the percentage of necrotic cells significantly decreased when HK-2 cells were pretreated respectively with inhibitors of RIP1/3 and MLKL. These data provide compelling evidence for a critical role of AngII in triggering the necroptosis of renal tubular epithelial cells in vivo and in vitro.

Apoptosis has been suggested to play a critical role in the loss of renal tubular epithelial cells in a previous study [33, 34]. In our previous study, Apoptotic renal tubular epithelial cells and cleaved caspase-3 expression were enhanced in kidney tissue from SNx rats, and this effect was inhibited by zVAD [5]. In the present study, we found a significant increase in the percentage of necroptotic cells among the HK-2 cells treated with 10^−9^ M AngII for 24 h using flow cytometric analysis and western blot analysis. However, we observed that the percentage of apoptotic cells was higher among the cells treated with 10^−7^ M AngII than among those treated with the other concentrations, as indicated by flow cytometric analysis. A possible explanation for this finding is that necroptotic HK-2 cells are more sensitive to AngII cytotoxicity than apoptotic cells. Therefore, we speculate that necroptosis might be an earlier and more important event in AngII-induced renal tubular epithelial cell death. In addition, the results indicate that necroptosis can switch to apoptosis as the AngII concentration increases and the blocking of apoptosis with zNAD can aggravate necroptotic cell death, in accordance with Galluzzi’s idea that apoptosis and necroptosis can occur simultaneously in cells and that necroptosis could even switch to apoptosis under special conditions [35]. However, we did not find that apoptosis and necroptosis occurred simultaneously in renal tubular epithelial cells in Ang II-induced chronic renal injury model 21 days post-implantation. Therefore, the relationship between apoptosis and necroptosis of tubular epithelial cells is not yet clear in vivo. We speculate that necroptosis and apoptosis might occur in different stages Ang II-induced chronic renal injury.

The exact mechanism by which AngII triggers RIP3 and MLKL activation in renal tubular epithelial cell necroptosis remains uncertain. In the present study, we found that AngII exposure significantly increased the expression of Fas and FasL protein and significantly elevated the percentage of necroptotic cells. Furthermore, disruption of AT1R and AT2R significantly reduced the levels of Fas and FasL protein in HK-2 cells, but inhibiting RIP1/3 and MLKL transfection with a specific small interfering (si) RNA did not decrease Fas and FasL expression. Importantly, blocking FasL significantly suppressed the percentage of necroptotic renal tubular epithelial cells. These data indicated that Fas and FasL were likely to be downstream target molecules of AngII and its receptors and might be upstream signaling molecules for RIP3 and MLKL. Therefore, we speculate that AngII mediates renal tubular cell necroptosis via its receptors and the Fas/FasL signaling pathway. A previous study showed that there was reciprocal expression between AT1R and AT2R in response to Ang II infusion, with evident AT1R upregulation and AT2R downregulation[36,37]. However, the current data clearly challenge that concept. At least in kidney tissue, Ang II-treated adult mice exhibited higher AT2R protein levels with increasing AT1R levels. Importantly, functionally antagonizing AT1R with losartan or blockading of AT2R activity with PD123319, could effectively diminish renal tubular epithelial cell necroptosis in Ang II-induced renal injury mice[38]. These results is similar to Ang II-induced apoptosis occurring via both AT1R and AT2R[39,40].

In conclusion, AngII cytotoxicity triggers necroptosis of renal tubular epithelial cells in vitro and in vivo, and secondarily increasedFas, FasL are involved in the AngII-induced regulation of cell death. Consequently, the therapeutic blockade of these molecules, for example, with the receptors of AngII and FasL inhibitors or with RIPK1/3 and MLKL blocking agents, can prevent AngII-induced excessive death of renal tubular epithelial cells in vitro and in vivo. The findings may correspond to a redundant mechanism of AngII exerting adverse effects on renal tubular cells in the initiation and progression of renal fibrosis and CKD and have potential clinical applications.

## Supporting information

S1 Fig Masson trichrome-stained mouse renal tubulointerstitial lesions in Ang II-induced chronic renal injury mice. Representative images of Masson trichrome-stained mouse renal tubulointerstitial lesions in AngII-treated mice treated with or without Nec-1 or zVAD. Masson-positive interstitial collagen regions were analyzed under a light microscope. Data represent the mean of three independent experiments ± S.E.M. N = 6 mice per group, ** p<0.01 compared with the control group, #p<0.05 compared with the vehicle group, ##p<0.01 compared with the vehicle group. According to the requirements of reviewers, S1 Fig has been inserted into Fig 1(D) and 1(F).
(TIF)

S2 Fig The ratio of annexin V+/PI- cells (apoptotic cells) numbers measurement in the HK-2 cells treated with AngII. The ratio of annexin V+/PI- cells (apoptotic cells) numbers was highest in the HK-2 cells treated with 10−7 M AngII (D) for 24 h. Data represent the mean of three independent experiments ± S.E.M. with n = 3, *p<0.05 compared with the control group, **p<0.01 compared with the control group. According to the requirements of reviewers, S2 Fig has been inserted into Fig 4(D).
(TIF)

S3 Fig Effect of zVAD on the percentage of necrotic HK-2 cells induced by Ang II. zVAD elevated the percentage of necrotic HK-2 cells induced by Ang II under TEM and confocal scanning laser microscope. Data represent the mean of three independent experiments ± S.E.M. with n = 3, *p<0.05 compared with the control group, **p<0.01 compared with the control group. To complete Fig 5, S3 Fig has been inserted into Fig 5.
(TIF)

S4 Fig We showed other 2 different samples per condition from other immunoblots experiments 2 times (S5 Fig, S4 Fig, S6 Fig, S7 Fig). (TIF)

S5 Fig We showed other 2 different samples per condition from other immunoblots experiments 2 times (S5 Fig, S4 Fig, S6 Fig, S7 Fig). (TIF)

S6 Fig We showed other 2 different samples per condition from other immunoblots experiments 2 times (S5 Fig, S4 Fig, S6 Fig, S7 Fig). (TIF)

S7 Fig We showed other 2 different samples per condition from other immunoblots experiments 2 times (S5 Fig, S4 Fig, S6 Fig, S7 Fig). (TIF)

S1 File Masson Trichrome staining analyses of renal tubulointerstitial injury. (DOCX)
