# Supplementary figures and images for: Angiotensin II triggers RIPK3-MLKL-mediated necroptosis by activating the Fas/FasL signaling pathway in renal tubular cells
Source: PLoS One. 2020 Mar 5;15(3):e0228385. doi: 10.1371/journal.pone.0228385 (PMC7058379; doi:10.1371/journal.pone.0228385)

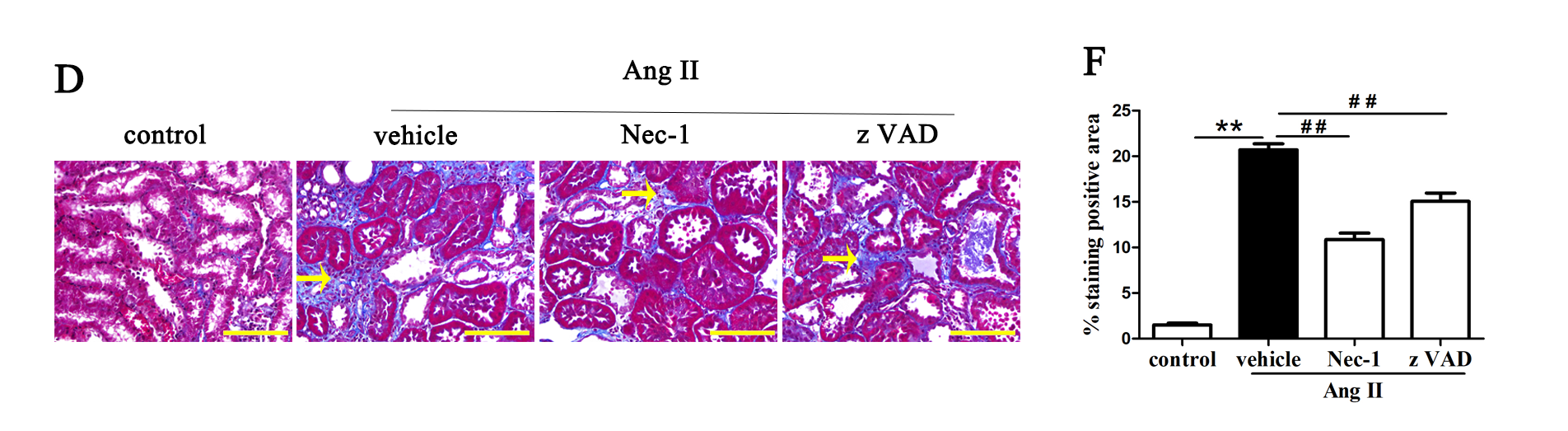

Supplement: S1 Fig — Representative images of Masson trichrome-stained mouse renal tubulointerstitial lesions in AngII-treated mice treated with or without Nec-1 or zVAD. Masson-positive interstitial collagen regions were analyzed under a light microscope. Data represent the mean of three independent experiments ± S.E.M. N = 6 mice per group, ** p<0.01 compared with the control group, #p<0.05 compared with the vehicle group, ##p<0.01 compared with the vehicle group. According to the requirements of reviewers, S1 Fig has been inserted into Fig 1(D) and 1(F). (TIF) [file pone.0228385.s001.tif]

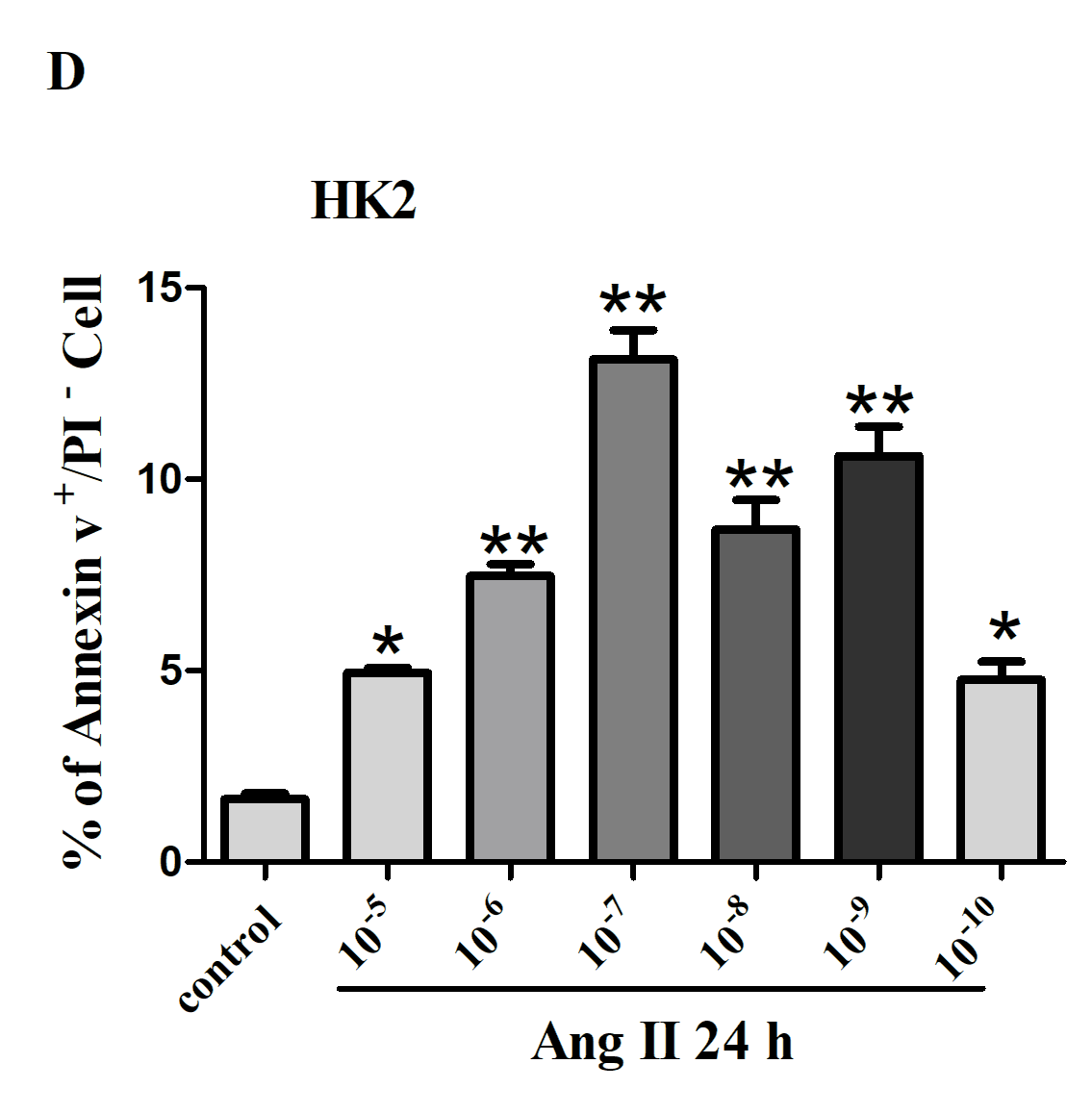

Supplement: S2 Fig — The ratio of annexin V+/PI- cells (apoptotic cells) numbers was highest in the HK-2 cells treated with 10−7 M AngII (D) for 24 h. Data represent the mean of three independent experiments ± S.E.M. with n = 3, *p<0.05 compared with the control group, **p<0.01 compared with the control group. According to the requirements of reviewers, S2 Fig has been inserted into Fig 4(D). (TIF) [file pone.0228385.s002.tif]

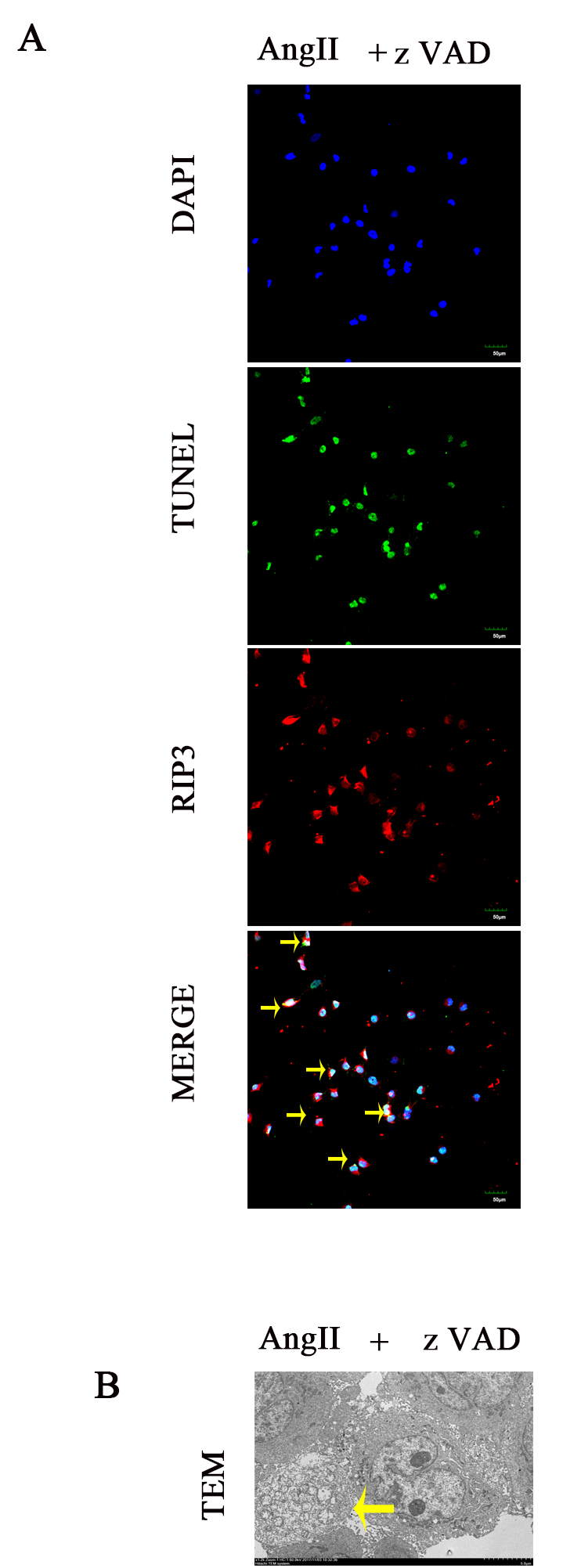

Supplement: S3 Fig — zVAD elevated the percentage of necrotic HK-2 cells induced by Ang II under TEM and confocal scanning laser microscope. Data represent the mean of three independent experiments ± S.E.M. with n = 3, *p<0.05 compared with the control group, **p<0.01 compared with the control group. To complete Fig 5, S3 Fig has been inserted into Fig 5. (TIF) [file pone.0228385.s003.tif]

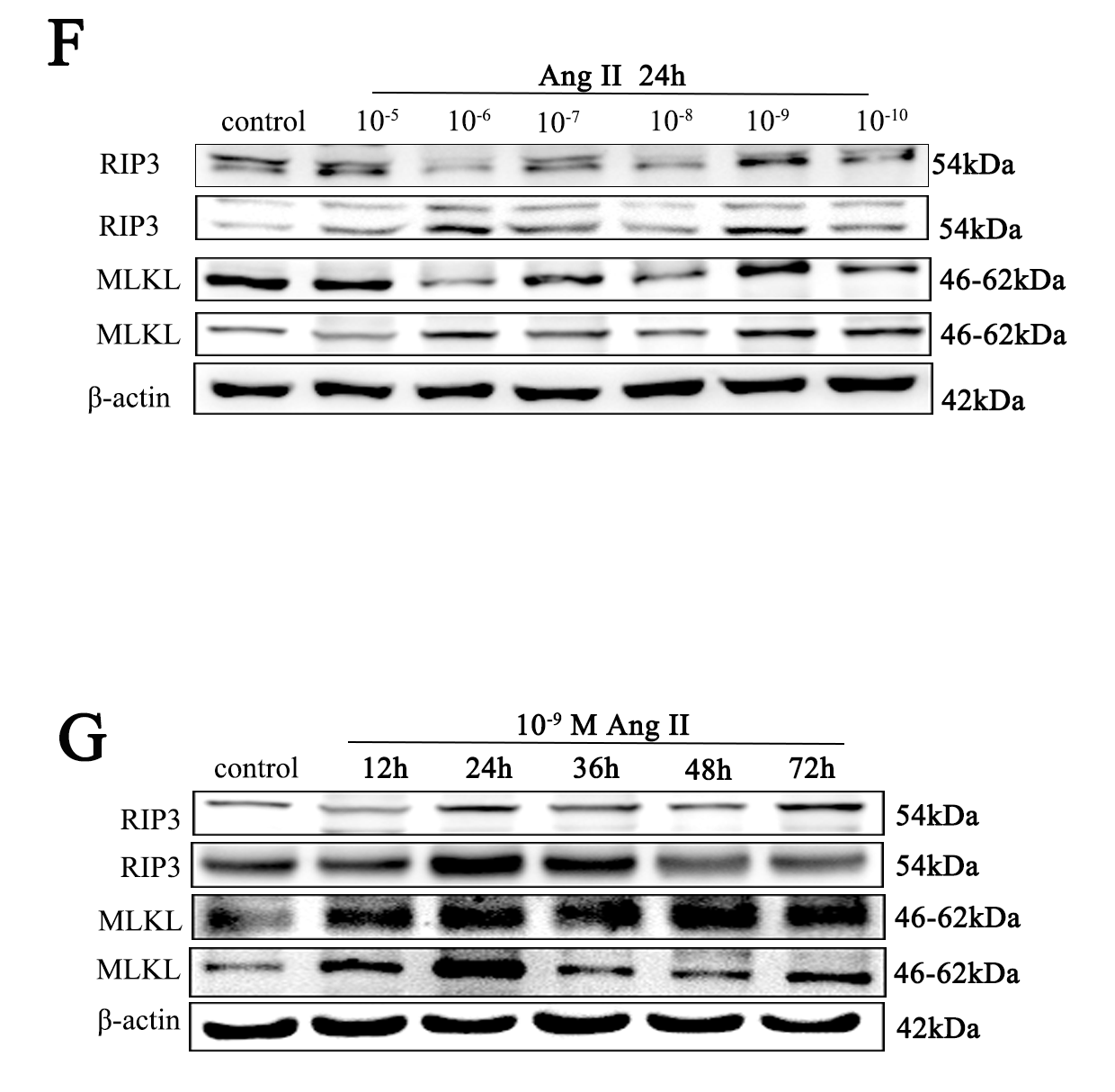

Supplement: S4 Fig — (TIF) [file pone.0228385.s004.tif]

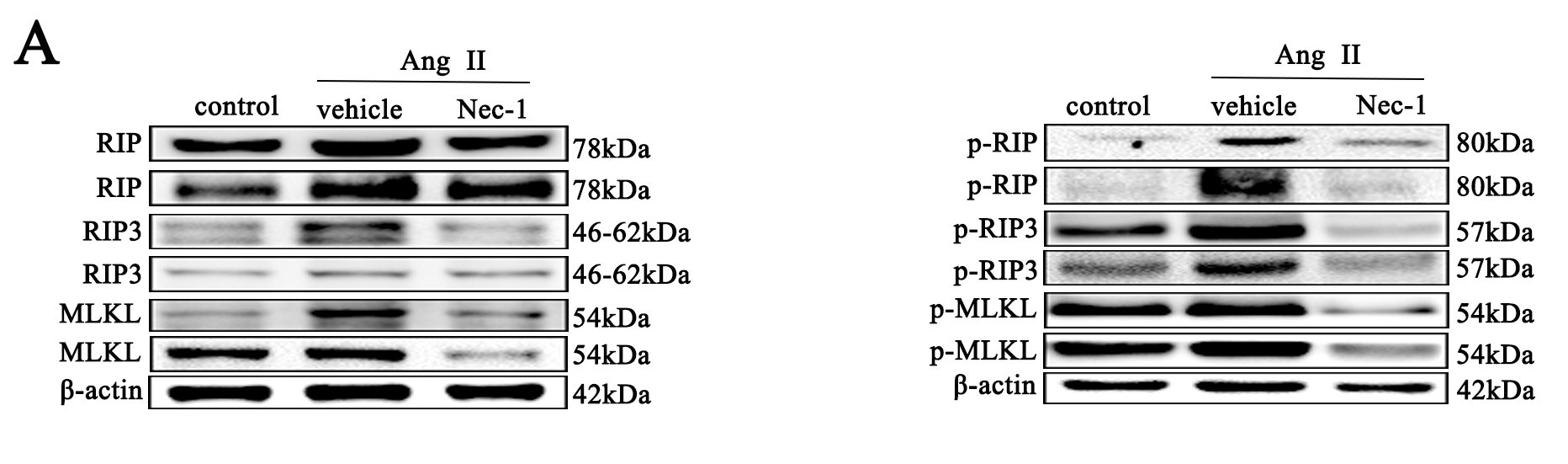

Supplement: S5 Fig — (TIF) [file pone.0228385.s005.tif]

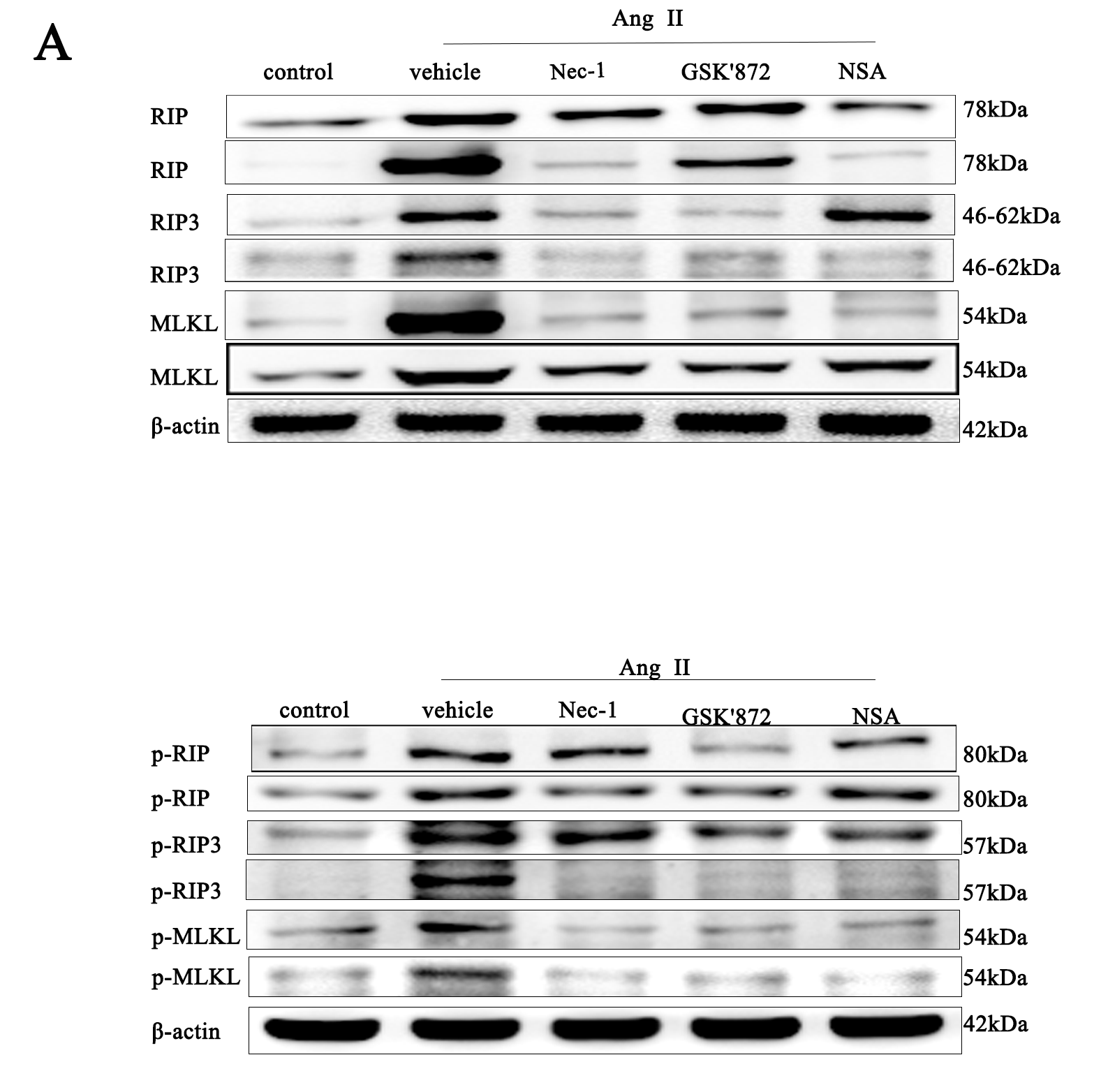

Supplement: S6 Fig — (TIF) [file pone.0228385.s006.tif]

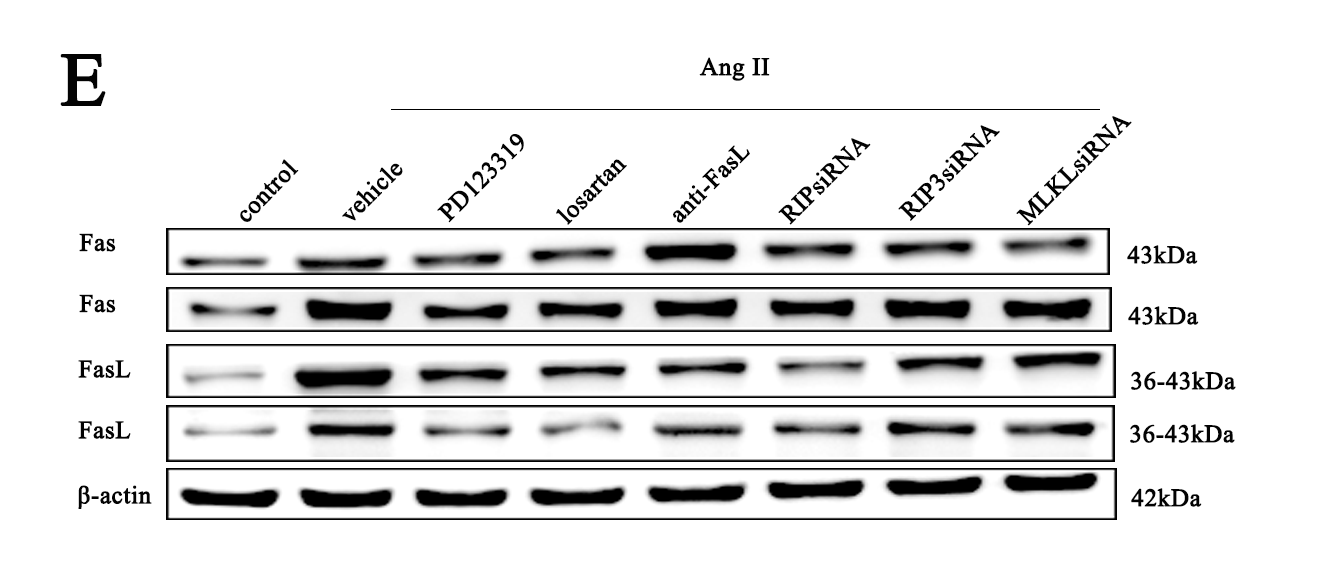

Supplement: S7 Fig — (TIF) [file pone.0228385.s007.tif]
