# Supplementary material for: Angiotensin II triggers RIPK3-MLKL-mediated necroptosis by activating the Fas/FasL signaling pathway in renal tubular cells
Source: PLoS One. 2020 Mar 5;15(3):e0228385. doi: 10.1371/journal.pone.0228385 (PMC7058379; doi:10.1371/journal.pone.0228385)
Supplement: S1 File — (DOCX) [file pone.0228385.s008.docx]

**Supporting Information**

**Masson Trichrome staining analyses of renal tubulointerstitial injury**

Kidneys of the mice were collected when the mice were sacrificed. One portion of the kidney tissue was fixed with 4% phosphate-buffered formaldehyde and embedded in paraffin wax. After deparaffinization and rehydration, kidney tissue sections were subjected to Masson Trichrome (Sigma-Aldrich) staining by following the manufacturers’instructions. The staining results were recorded under a bright field microscope. For the quantitative analysis, at least 10 random high-power fields (i.e., 400x) were selected and evaluated. The positive area was calculated as a % of the total area. All assessments were performed in a blinded manner by an experienced pathologist.
